# South Asians living in the UK and adherence to coronary heart disease medication: a mixed- method study

**DOI:** 10.1007/s11096-018-0760-3

**Published:** 2018-12-18

**Authors:** Zahraa Jalal, Sotiris Antoniou, David Taylor, Vibhu Paudyal, Katherine Finlay, Felicity Smith

**Affiliations:** 1 School of Pharmacy, University of Birmingham, Edgbaston, Birmingham, B15 2TT UK; 2 Pharmacy Department, Barts Heart Centre, Barts Health NHS Trust, London, UK; 3 Department of Practice and Policy, School of Pharmacy, University College London, London, UK; 4 Department of Psychology, The University of Buckingham, Buckingham, UK

**Keywords:** Adherence, Cardiovascular medication, Coronary heart disease, South Asians, United Kingdom

## Abstract

*Background* The prevalence of coronary heart disease amongst South Asian population in the UK is higher compared to the general population. *Objective* This study sought to investigate beliefs and experiences of South Asian patients regarding coronary heart disease and medication taking behaviour. *Setting* A London Heart Attack Centre. *Methods* This mixed method study is part of an original pilot randomised study on 71 patients involving a pharmacy-led intervention to improve medication adherence in coronary heart disease patients. South Asian patients from the randomised study took part in qualitative semi-structured telephone interviews. Both South Asian and non-South Asian patients completed the questionnaire about adherence and beliefs regarding medicines using Morisky Scale and the Belief About Medicines Questionnaire-Specific at 2 weeks, 3 and 6 months. *Outcome* Patients’ beliefs about coronary heart disease and medication adherence. *Results* Seventeen South Asian patients and 54 non-South Asian patients took part. Qualitative data from 14 South Asian patients showed that while some attributed coronary heart disease to genetic, family history for their illness, others attributed it to their dietary patterns and ‘god’s will’ and that little could be done to prevent further episodes of coronary heart disease. On the Belief About Medicines Questionnaire-Specific in South Asian patients, beliefs about necessity of medicines outweighed concerns. South Asian patients (n = 17) showed a similar pattern of adherence compared to non-Asian patients (n = 54). Adherence decreased with time in both populations, adherence measured by Morisky Scale. *Conclusion* South Asian patients in this study often attributed coronary heart disease to additional causes besides the known risk factors. Future studies on their understanding of the importance of cultural context in their attitudes to prevention and lived experience of the disease is warranted.

**Electronic supplementary material:**

The online version of this article (10.1007/s11096-018-0760-3) contains supplementary material, which is available to authorized users.

## Impacts on practice

- There is a need to promote both primary and secondary prevention interventions for adherence to medication in UK South Asian patients with coronary heart disease.
- There remains a need for education within the South Asian communities on the causes and prevention of coronary heart disease.

## Introduction

Coronary heart disease remains the most common cause of premature death in the UK. In 2014, 15% of male deaths and 10% of female deaths were from CHD, a total of around 69,000 deaths [1]. South Asians (SAs) living in the UK (people from India, Pakistan, Bangladesh, Sri Lanka and Nepal) have a higher death rate from CHD at a younger age, often before the age of 40 years in men, 40% higher than the whole population [2]. It is important to mention that SAs are heterogeneous in relation to culture and risk factors for heart disease [3]. An explanation for excess deaths from CHD in SAs is still unclear. Several theories in previous literature include migration, disadvantaged socioeconomic status, proatherogenic diet, lack of exercise, high levels of homocysteine and LP(a) lipoprotein, endothelial dysfunction, enhanced plaque and systemic inflammation [4]. Other studies attribute this to the fact that SAs have substantially higher rates of diabetes, a risk factor often linked to higher CHD mortality [5]. People who have had a myocardial infarction (MI) benefit from treatment to reduce the risk of further manifestations of vascular disease; this is known as secondary prevention. Secondary prevention after an acute MI includes cardiac rehabilitation, lifestyle changes and drug therapy. The use of secondary prevention medication in patients after CHD is vital to maintain optimal heart function [6]. Research has shown that consistent use of medication after a coronary event is associated with lower adjusted mortality and higher survival rates [7, 8]. Cessation of secondary prevention medications can lead to rehospitalisation and re-infarction and increased risk of death [9, 10]. However, non-adherence rates to medication for MI patients range from 13 to 60% [9]. A systematic review of 17 studies [11] conducted in India, Pakistan and Sri Lanka investigated adherence rates to cardiovascular medication in SA patients. The review showed low rates for adherence of only 32% and concluded that there is a need for future interventions to target adherence in this vulnerable population, where premature cardiovascular disease is a growing epidemic [11]. There has been little research on adherence to coronary heart disease medication in SA patients living in the UK, despite the relatively high prevalence of cardiovascular conditions in this group [6]. It is not clear if ethnicity plays a role in medication non adherence [12], only few studies have been conducted [11, 12] and have shown that adherence to cardiovascular medication is low when compared to non-Asian counterparts [12].

There is a dearth of literature on how personal beliefs shape the way that medicines prescribed for the treatment of cardiovascular disease are taken by people of South Asian origin [13, 14]. There is hence a need to identify health beliefs that may contribute to health behaviours in South Asian patients with elevated risk of lifestyle-related disease [14]. Therefore, there is a need to look closer at this particular ethnic group regarding adherence and beliefs around medication after a CHD.

## Aim of the study

To investigate the UK South Asian patients’ beliefs and lived early experiences about coronary heart diases and their beliefst, experiences and behaviours relating to medication taking in CHD.

## Ethics approval

Ethics approval for the interviews was gained from National Research Ethics Service Committee North West –Preston, also from the R &D Joint Research Management Office Queen Mary Innovation Centre and the R&D office University College of London.

## Methods

This was a mixed method study including qualitative in-depth interviews followed by questionnaires. It was conducted as part of an original pilot study [15] on 71 patients (54 non-Asians and 17 SAs) that assessed the impact of a pharmacy-led intervention to improve adherence amongst CHD patients. As an approach to focus on adherence to cardiac medication in only SA patients, 17 patients were invited to participate in a telephone interview, 3 months after hospital discharge.

Sampling and recruitment for the qualitative interviews: Eligibility criteria included 1-patients who have had a coronary event unstable angina, ST elevation myocardial infarction or non ST elevation myocardial infarction, 2-were prescribed secondary prevention medication, 3-were from SA origin, 4-could communicate in English and 5-had signed written consent to be part of the pilot study. These patients were approached before discharge by the researcher. This is to confirm if they would be interested in participating in telephone interviews. Seventeen patients matched the eligibility criteria and were invited to the interviews. Fourteen SA patients agreed. Ethnicity was determined from the patients’ hospital charts and further confirmed during the recruitment process.

### Study setting

The study was undertaken in collaboration with a London Heart Attack Centre in a district in East London UK.

### Data collection

Quantitative data on medication adherence: The self-report 8-Item Morisky Medication Adherence Scale (MMAS-8) was used to assess adherence [16]. The MMAS-8 has a high reliability, sensitivity and specificity and has been widely used in research on medication adherence. The MMAS-8was measured at baseline (2 weeks after discharge) and at 3 and 6 months. The Belief about Medicines Questionnaire Specific (BMQ-S) was used to assess patients’ beliefs about their medication. It is a valid and reliable scale having been validated for use across a range of different diseases and also cardiac illnesses [17]. The BMQ specific is comprised of two subscales, which are BMQ necessity and BMQ concerns. The BMQ specific was measured at 3 months.

Qualitative data: The questions were developed by the research team and adapted from previous literature [9]. Previous literature has shown that factors that influence medication-taking behaviour in coronary heart disease include perception about both the disease and medicines [18].

### Data processing and analysis

Data from the Morisky and BMQ-scale were analysed using SPSS Version 22 and linked with the results from the interviews. The BMQ specific scale is comprised of two subscales, which are BMQ necessity and BMQ concerns. The scores of each subscale are computed from the sum of all items within that particular subscale and range from 5 to 25. The necessity-concerns differential can be computed by subtracting the total BMQ concerns subscale score from the total BMQ necessity subscale score. A positive differential score indicates that the participants perceive the benefits of their medication to outweigh the risks, in contrast negative differential score indicates that participants perceive the risk of taking their medication outweigh their benefits. The differential scores range from − 20 to 20 [17].

All interviews were transcribed verbatim. A framework approach to analysis was undertaken involving the development of an initial coding framework with each domain of interview schedule, informed by literature and themes that emerged from the data. As analysis proceeded this was modified and refined using constant comparison techniques; in which all items of data assigned a particular code, were appraised for similarities and divergences from those already coded. To ensure reliability of the coding procedure, coding of the transcripts was undertaken by two members of the research team.

## Results

A total of 71 patients took part in the study; 54 (76.0%) were males; 51 (71.8%) had experienced ST-elevation MI and 20 (28.2%) non-ST elevation MI. Of these 17 (23.9%) were SA patients. Majority were in the age bracket of 60–69 (n = 20) and 70-79 (n = 20).

### Belief about medicines questionnaire

A total of 14 of the 17 SA patients completed the BMQ. Of these 11 patients scored positive on the BMQ-S and 3/14 had a negative score. In BMQ-S the 14 SA patients had a mean (± SD) necessity score of 21 and a mean (SD) concern score of 15 (Table 1). This showed that more patients at 3 months believed that the benefit of their medicines outweighed their concerns. Patients who had positive BMQ-S scores had a score that was positively linked with adherence (Table 1).

**Table 1:** Results on the belief about medicines questionnaire specific

Patient no.	Control	Intervention	Result on BMQ	Adherence baseline, 3 months 6 months		
1	BMQ 25/10		Positive	8	8	8
2	BMQ 25/19		Positive	8	8	8
3		BMQ 25/12	Positive	6.75	8	7
4		BMQ 25/12	Positive	7.5	8	7.5
5		BMQ 21/15	Positive	8	8	8
6		BMQ 24/11	Positive	8	8	8
7		BMQ 17/21	Negative	5.75	5.75	5.75
8		BMQ 20/19	Positive	8	8	8
9		BMQ 21/15	Positive	8	8	8
10		BMQ 15/20	Negative	5.75	0.7	0
11	BMQ 19/10		Negative	7	7	8
12		BMQ 19/14	Positive	7	6.75	7
13	BMQ 20/13		Positive	8	8	5.5
14	BMQ 21/16		Positive	8	8	6.75
			21/15	Mean score		

A positive differential score indicates that the participants perceive the benefits of their medication to outweigh the risks, in contrast negative differential score indicates that participants perceive the risk of taking their medication outweigh their benefits

### Adherence

**Fig. 1:**
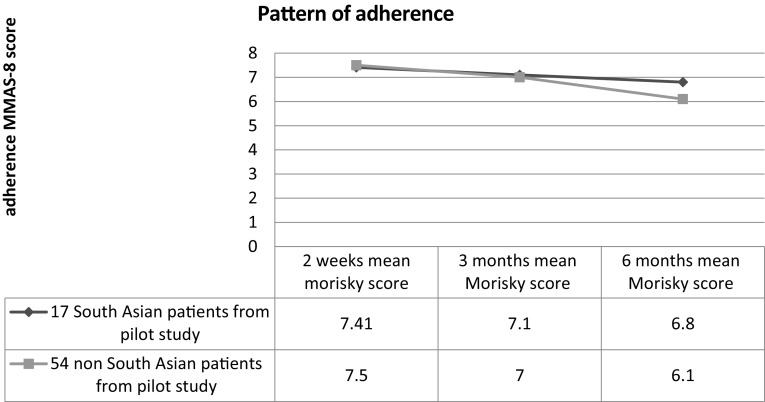
Patterns of adherence. *Adherent patients (Morisky score moderate 6–8, Morisky score high = 8) and patients with suboptimal adherence (Morisky score < 6). *The pilot study included 71 patient 54 non-South Asian and 17 South Asian patients, 14/17 South Asian patients’ took part in the qualitative interviews

All 71 patients including 17 SA patients and 54 non-SA patients completed the 8-Item Morisky Scale (MMAS-8) at baseline. The SA patients’ had a mean (SD) adherence MMAS-8 scale of 7.41 from 8 and this decreased with time to 7.1 and 6.8 at 3 and 6 months respectively (Fig. 1). A similar pattern of adherence to medication was observed in the non-SA patients from the pilot study (non-Asian) adherence to medication decreased with time 7.5 at baseline and this decreased to 7 and 6.1 at 3 and 6 months respectively. Statistical analysis showed that there was no evident difference in adherence patterns between the SAs’ and the patients in the pilot study after an acute myocardial infarction as shown in the figure below.

### Qualitative interviews

Fourteen interviews were carried out between January 2014 and April 2014. The interviews lasted around 20 minutes. Participants’ ages ranged from 32 to 72 years with 7 patients in their 30’s and 40’s. Thirteen were male and one only was a female, country of origin of patients included Bangladesh (N = 5), Pakistan (N = 3) and India (N = 6). Three main themes were derived from the results: perception of patients regarding their disease, perception regarding their medication and factors that influenced adherence to secondary prevention medication, in addition sub-themes emerged from the data.

### Theme 1 perception of patients regarding their disease

#### South Asian patients’ beliefs and lived early experiences about CHD

##### Beliefs regarding the disease feeling ill versus healthy

South Asian patients described their experience of a heart attack as being intense chest pain accompanied by regular symptoms of a heart attack, severe burning pain and vomiting. Few patients described it as less intense, one patient believed he had a breathing problem and another described it as a vomiting problem.

I had a breathing problem heavily too much and now I am walking no breathing problem (male 68 years of Indian origin participant no. 1).

Some relatively older patients in this sample believed that the disease was acute and after they had a procedure they were cured and therefore questioned the necessity of using medicine for a long period.

The disease is not serious now, I am not sure whether I need medicines and should I continue medicines or not? (68 years, male of Indian origin participant no. 1).

When considering the Morisky scale for these patients it was observed that they had low scores (e.g. BMQ negative score, Morisky scale 5.75 at baseline, 3, 6 months- Fig. 1). Other patients found it difficult to accept the disease.

I was healthy and I didn’t take medicine all my life and then suddenly you have 6 different medicines a day this is a confusion (32 years old male from Bangladesh participant no. 3).

### Causes of the disease

Patients in the interviews reported that the main cause of their disease was attributed to genetics and family history and that the disease was inevitable and running in families.

My family history my father died only when he was 51 and my brother when he was only 31 and he died he had another heart attack (male from Bangladesh participant no. 12).

May be my family, it is running in my family, once you have it you have it (male from Bangladesh participant no. 3)

Some patients mentioned that the cause could be related to their food which was described as containing a lot of rice and red meat and also the use of ghee for cooking.

It is our food, we Asians we eat a lot of curry and rice and greasy food (male from Bangladesh participant no. 14).

The younger patients in this sample linked the importance of healthy lifestyle choices for disease prevention.

I was going to the gym and I always look carefully at what I eat and I cut down on my alcohol (Patient of Pakistani origin age 48 participant no. 4).

I exercise nearly every day, I do this all the time, every day I have to go out for a good walk (43 year-old patient from Indian origin participant no. 7).

Other reported causes from the interviews included stress and a flu after going for Hajj.

I am stressed from my work and it is stress that caused my heart attack but God gave me the life (52 years old female of Pakistani origin participant no. 2).

Before I was healthy but then I had a flu after back from Hajj, this is what it is (72 years old Male Pakistani origin participant no. 10).

Fatality: some patients mentioned the idea of the will of God and not the individual to determine future health.

What can you do I mean if God gave you the life whatever he gave, you have to live, it is in God’s hands (Female of Pakistani origin participant no. 2).

Worries of having a second heart attack were raised in more than half of the patients’ interviews.

Now I worry all the time, it could happen any time again (male of Pakistani origin participant no. 10).

Some patients in this sample believed that the medicines might be able to prevent recurrence of CHD, these patients had positive BMQ-S scores.

### Theme 2 perception of patients regarding their medicines

#### South Asians’ beliefs, experiences and behaviours relating to medication taking in CHD

##### Role of medication necessity verses concerns

The majority of patients perceived the medicines to be very important for their current health and only few where not sure. However, some patients expressed that the number of tablets to take was high and this led to additional concern regarding medicine safety.

I think they are pretty important that is the reason they gave them to me but too much medicine, is this good for you? (male of Indian origin participant no. 8.)

### Knowledge regarding the medicines

Half of the patients in this sample were able to list what their medicines were for. On the other hand half reported little knowledge, mostly relying on family members or on a healthcare professional or simply not interested in knowing.

They are for cholesterol and clotting something like that, do not really know (male of Bangladeshi origin participant no. 5).

Chemist explained but memory not good (male of Bangladeshi origin participant no. 9).

Yes my GP told my son and at the hospital they told my son this is for this and this is for that, but they are important to take (Female Pakistani origin participant no. 2).

### Theme 3 factors that influenced adherence to secondary prevention medication

#### Factors derived from the interviews that influenced adherence in interviewed South Asian patients

##### Forgetfulness

Several patients had problems with remembering to take their medications. The patients reported that this was attributed to being on medication for the first time, their age, or trying to fit the medication into a habit as a daily routine.

I take the medication all once a day so if I forget I forget the whole lot (Bangladeshi patient aged 42 participant no. 12).

These patients were young patients and reported that their non-adherence was unintentional. They expressed a need to accept the disease and adapt to a new life style and routine that would include medication taking. Ways to remember to take medicines were reported as use of a pill box, writing on the boxes in native language, arranging them in a bag and making them accessible around the house.

### Family support

Family support varied among the patients, support was either offered as help in organizing the medicines, providing the knowledge about the medicines, helping with healthy eating and stress management. The patients’ reported that family support they received helped with their adherence to the medicines.

My sister and my cousin they are trying to help me, my daughter gives me the medicine and I just take so I remember (male of Indian origin participant no. 6).

### Side effects

The main side effects reported by the patients included muscle pain, coughing, cold extremities, bruising and lethargy. The patients who experienced side effects reported in the interviews that they continued to take their medicines.

One tablet ramipril tablet I was coughing and the coughing comes and goes every time, I told my GP and now I am on a different medication and I take it every day (male of Pakistani origin participant no. participant no. 10).

My feet do feel cold then they do not stay long it is just you know, it is not a major thing I still take my medicine (Female of Pakistani origin participant no. 2).

### Relying on health care practitioner

More than half of the patients relied on a healthcare practitioner regarding the decision to take medicines.

The doctor said they are important for my body, I do not think about it if doctor says I need it then I need it that’s it (male of Indian origin participant no. 11).

### Living in areas of deprivation

Two patients reported living in areas of deprivation (bad accommodation, large families, drugs and depressing environments) which affected healthy dietary choices and added further stress to their physical and mental health. This was accompanied by depression and had a negative effect on adherence to medicines after a CHD.

I have been living in this hostel for 4 years and people in this place are too many they are always banging shouting and screaming, I cook my food and if I leave it for 10 min it is gone from the cooker, I need to get out of this hole (male of Bangladeshi origin participant no. 5) BMQ Negative score, Morisky scale 5.75, 0.7, 0 at baseline, 3, 6 months).

### Cardiac rehabilitation

All the patients had either attended or were committed to attending cardiac rehabilitation, except one patient that thought that cardiac rehabilitation was further injections and procedures.

No I didn’t go to cardiac rehabilitation. If they start putting injections again, I do not want to go through that again (male of Bangladeshi origin participant no. 3).

The patients’ who attended cardiac rehabilitation reported that it was very useful and helpful.

I went to cardiac rehabilitation and I am thinking right now of joining a gym, I have changed the food I eat after cardiac rehabilitation (male of Bangladeshi origin participant no. 5).

## Discussion

### Key findings

The results illustrate that there was a belief in the importance of the medicines after a coronary event. The patients who adhered to the medicines believed that the medicines could prevent future events. Older patients perceived the disease to be of an acute nature and were unable to recognise unhealthy life style as a risk for CHD. In contrast younger patients had difficulty in accepting the disease and this affected their ability to adapt to a routine for medication taking; but were more aware of risk factors for CHD. CHD is perceived as a disease of senior citizens, as only 4–10% of all MIs occur before age 45 [19]. However, SA patients more often experience CHD before the age of 40; and in this sample half of the patients were in their 30’s and 40’s. Patients reported in the interviews that family history and genetics were the main cause of their disease, also that the disease was inevitable and thus early prevention and further treatment might not have a significant role. Moreover, patients expressed strong family support with their disease and medication.

This study included a small sample size. Nevertheless a similar pattern of adherence to medication was observed in the SA patients compared to the non-SA patients (n = 54) from the pilot study. Adherence reduced with time in both groups. The SA participants’ in this study expressed the importance of attending cardiac rehabilitation and had attended or were scheduled to attend. Previous literature has shown that low-levels of cardiac rehabilitation participation among SA and other ethnic minority groups have been reported in several English-speaking countries including the USA, Canada, the UK and Australia [20]. This has been attributed to reasons such as exercise, culture and religion, programme access and structure and communication and language [21]. In the UK, the key health policy outlining the national standard for cardiac rehabilitation, the National Service Framework (NSF) for Coronary Heart Disease [22], states that services should be accessible and acceptable to all the people they serve regardless of their ethnicity. This includes services able to meet people’s needs in ways that are culturally, religiously and linguistically appropriate [23]. Several UK-based community health projects designed to improve CHD prevention and rehabilitation among SA individuals, such as Project Dil in Leicester [24], the BRUM study in Birmingham [25] and the Khush Dil project in Edinburgh [10], offer encouraging directions for healthcare professionals, in the design and delivery of culturally sensitive cardiac rehabilitation services [21].

The findings in this study should be approached with caution due to the small sample size and the short length of the interviews. Nevertheless, this study showed that increased belief regarding the necessity of the medicines was associated with positive adherence scores. This finding is in line with other studies with large sample sizes, for example a study that involved 1611 coronary heart disease patients from 35 practices in Ireland found; that a strong belief in the necessity of one’s medication and a lower level of concern about one’s medication were related with higher levels of adherence [26].

Studies in patients with chronic diseases have previously shown that medication adherence can be improved if patients are provided with information regarding the prescribed medicines [27]. Factors that influenced adherence in our study included: forgetfulness, depression, side effects, living in areas of deprivation. These factors fall under the WHO framework for medication adherence that lists reasons for non-adherence to include younger age, socioeconomic factors, mental health (depression) and side effects [28] and can also be compared to results of a review [6] that concluded that medication side-effects, knowledge regarding the medicines, cost, forgetfulness and higher frequency of dosing are contributed to non-adherence.

The patients expressed in interviews that they follow doctors’ orders and instructions with the belief that health management should be left to qualified health professionals. This has also been concluded in other studies examining health beliefs of UK SAs’ related to chronic diseases [29].

SA population in our study were aware of risk factors for CHD, however, the older participants in this sample seemed less aware of the general facts about CHD. Previous published literature has shown that awareness may be high in the general population regarding CHD compared to other diseases. The UK’s 2007 National Stroke Strategy for England (NSSFE) showed that the general population awareness is high for coronary heart disease but lower for stroke [30]. A further study in 2010 agreed with the NSSFE view, which showed there was more awareness of general facts in the population about CHD than stroke [30]. Nevertheless, a previous cross-sectional UK study including 334 SA men and women aged 16–74 years showed that for both heart disease and diabetes, two-thirds of respondents said they did not understand enough about the conditions to prevent them [31]. In interviews 35% of people said they did not understand the meaning of the term heart disease, 14% could not give a single cause, and 17% could not suggest a preventive measure. [31].

### Limitations

In this study patients unable to understand and communicate in English were not included, this was a restriction to understanding communication barriers between SA patients and health care practitioners and how this could reflect on adherence. Other limitations include imbalanced gender mostly male and only one female took part in the interviews, therefore the view might not be representative. Furthermore, telephone interview has drawbacks against face-to-face interview: e.g. not able to build rapport with participants, people might be less likely to talk on the phone, which subsequently did not generate rich data.

### Recommendations for practice and research

Due to the small number of participants in our study a larger qualitative study focusing on SA uptake of programmes such as cardiac rehabilitation would be of benefit. Emphasis on primary prevention in high risk SAs needs to be prioritised, this can be achieved by education about the excess risk and risk factors and by campaigns targeted and tailored towards SA communities living in the UK.

## Conclusion

This article sheds light on secondary prevention after a myocardial infarction in SA patients, in regards to behaviour, adherence to medicine, living with the disease and cardiac rehabilitation. Similar adherence patterns to coronary heart disease, secondary prevention medication in SA patients and the non-Asian patients included in this sample. However, older SA participants in this study perceived the disease to be of an acute nature, in contrast younger patients despite being more aware of the facts and risk factors for CHD, had difficulty in accepting the disease and this affected their ability to adapt to a routine for medication taking. There are very few studies focusing on beliefs and adherence patterns after coronary heart disease of SAs in the UK and due to the large SA population in the UK, larger studies of this nature are imperative.

## Electronic supplementary material

Below is the link to the electronic supplementary material.

Supplementary material 1 (DOCX 13 kb)
