# Supplementary material for: South Asians living in the UK and adherence to coronary heart disease medication: a mixed- method study
Source: Int J Clin Pharm. 2018 Dec 18;41(1):122–30. doi: 10.1007/s11096-018-0760-3 (PMC6394505; doi:10.1007/s11096-018-0760-3)
Supplement: Supplementary file 1 — Supplementary material 1 (DOCX 13 kb) [file 11096_2018_760_MOESM1_ESM.docx]

Supplementary material

The semi structured interview guide for the study- South Asians living in the UK and adherence to coronary heart disease medication: a mixed- method pilot study:

| The semi structured interview adapted from Garavalia et al, 2009 *  First could I ask you to describe your heart attack that led to your hospitalization?  Could you tell me your thoughts about the medications that you have been prescribed?  Prompts: Do you believe they are important? Could you share any specific thoughts and beliefs you may have about your medicines.  Could you tell me how important you currently think your medicines for your heart disease are?  Please could you describe your everyday medication taking:  Prompts: if you are having problems in taking your medication?  For example are you using a pill organiser? Are you experiencing side effects? Are you having any problems in forgetting to take your medicines?  Could you tell me what your medications are supposed to do?  At this point, what do you think about your heart disease or do you think your heart disease is a serious matter?  Could you please describe your lifestyle before your heart attack?  Prompts: Do you exercise, go to a gym? What kind of exercise do you often undertake if any? Your diet? Smoking? Alcohol intake.  Could you please describe any life style changes or daily routines you might have made after your heart disease/attack?  How do you think your heart disease affects your life or may change your future? |
| --- |
| * Garavalia L, Garavalia B, Spertus JA, Decker C. Exploring Patients’ Reasons for Discontinuance of Heart Medications. J Cardiovasc Nurs. 2009; 24(5): 371–379.  The questions for the semi-structured interviews were adapted from a previous qualitative descriptive study Garavalia et al, 2009, that investigated coronary heart disease patients’ perspectives and beliefs regarding their medication. The questions were modified and three domains were studied: perception of disease, perception about the medication and factors that influence adherence. The questions were piloted with 2 patients before conducting the interviews. Pilot data was not included in the results. |
